# Enhancement or Reduction of Sonochemical Activity of Pulsed Ultrasound Compared to Continuous Ultrasound at 20 kHz?

**DOI:** 10.3390/molecules18054858

**Published:** 2013-04-24

**Authors:** Yujing Sun, Xingqian Ye

**Affiliations:** 1 Department of Food Science and Nutrition, School of Biosystems Engineering and Food Science, Zhejiang University, Hangzhou 310058, China; 2 Fuli Institute of Food Science, Zhejiang University, Hangzhou 310058, China

**Keywords:** pulsed ultrasound, continuous ultrasound, sonochemical activity, power ultrasound

## Abstract

Little is known about the efficacy of pulsed ultrasound compared with continuous ultrasound. Previous studies on the efficacy of pulsed ultrasound were not systematic and gave different results. In this study, the effects of pulse length, pulse interval, pulse length × pulse intervals, and treatment time on sonochemical activity were investigated using a simple oxidation of iodide method and a comparison of the efficacy of pulsed ultrasound and continuous ultrasound is made. The results showed that the main factor in the efficacy of pulsed ultrasound was pulse length when pulse length varied from 0.1 to 1 s. However, the main factors were pulse length, the pulse length × pulse interval, and pulse interval when pulse length varied from 1 to 9 s. Pulsed ultrasound had no effect when the pulse length was 0.1 s; however, the sonochemical activity of pulsed ultrasound decreased compared to continuous ultrasound as the pulse length varied from 0.1 to 1 s. The sonochemical activity of pulsed ultrasound either increased or decreased compared to continuous ultrasound when pulse length varied from 1 to 9 s, but the increase or decrease had no clear trend. The sonochemical activity was constant at T_on_/T_off_ = 2 s/2 s and slightly decreased at T_on_/T_off_ = 3 s/2 s with time, whereas the sonochemical activity of continuous ultrasound significantly decreased with time. Enhancement or reduction of sonochemical activity of pulsed ultrasound compared to continuous ultrasound depended on the pulse length and pulse interval.

## 1. Introduction

Power ultrasound can be used in various processes of the food industry, including processing [1,2,3], preservation [4], elimination of pesticides [5,6], extraction of bioactive compounds [7], preparation of microencapsulated food [8], and novel foods [9]. Of course, ultrasound is still under research in the lab for most of the processes mentioned above. Using ultrasound, food processes can now be completed in a way that reduces processing costs, simplifies manipulation, and consumes only a fraction of the time normally needed for conventional processes. The higher efficiency of ultrasound than conventional processes can mainly be attributed to the acoustic intensity. Acoustic intensity measurement methods are based on the heat, mechanical, optical and chemical effects produced by ultrasonic cavitation [10,11,12], and the chemical effect of ultrasound is a simple measurement of acoustic intensity. In this paper the ultrasound efficiency was expressed by sonochemical activity. 

Most studies on the application of ultrasound in food processing were carried out under continuous mode, with little research performed under pulsed mode. Adekunte [13] studied the effects of pulsed ultrasound on the color, ascorbic acid content, and yeast inactivation of tomato juice under conditions of T_on_/T_off_ = 5 s/5 s and a constant frequency of 20 kHz. Zúñiga *et al*. [9] prepared aerated gelatin gels stabilized by whey protein β-lactoglobulin using pulsed ultrasound under conditions of T_on_/T_off_ = 0.3 s/0.7 s and frequency varying from 20 kHz to 100 kHz. Valdramidis *et al*. [14] analyzed ascorbic acid degradation and non-enzymatic browning of orange juice during pulsed ultrasound processing under conditions of T_on_/T_off_ = 5 s/5 s at a constant frequency of 20 kHz. Rodríguez-Rojo *et al*. [15] extracted rosemary antioxidants with green solvent under conditions of T_on_/T_off_ = 30 s/30 s at a constant frequency of 20 kHz. However, these studies were carried out under limited pulsing conditions; relatively little research has been performed regarding the efficiency, the rules governing the changes in sonochemical activity, and the mechanism of pulsed ultrasound in food processes. 

Although few food scientists have paid attention to the efficacy of pulsed ultrasound, some chemical scientists have done work on the sonochemical activity of pulsed ultrasound under limited conditions. However, these studies have not presented consistent results. Some researchers found that sonochemical activity decreased under pulsed ultrasound compared to continuous ultrasound [16,17], while some researchers [18,19] found that sonochemical activity was enhanced under pulsed ultrasound, and other researchers [20] found that there was no clear trend.

The objective of this study was to analyze the effects of different factors on sonochemical activity under pulsed ultrasound, comparing the effect of pulsed ultrasound with that of continuous ultrasound using a simple iodide oxidation method. The results will help to gain a clearer understanding of efficacy of pulsed ultrasound in food processing and in other chemical processes.

## 2. Results and Discussion

### 2.1. Effects of Pulse Length and Pulse Interval

The effects of pulse length and pulse interval on the pulse enhancement (PE), which is defined by Equation (2) in the Experimental section, are shown in Table 1, Table 2. The results indicated that the sonochemical activity of pulsed ultrasound decreased compared to that of continuous ultrasound as the pulse length increased from 0.1 to 1 s. When the pulse length increased from 1 to 9 s, the sonochemical activity of the pulsed ultrasound decreased compared to that of continuous ultrasound when the duty cycle was less than 50% and either increased or decreased when the duty cycle was greater than 50%. However, the increase or decrease of sonochemical activity under pulsed ultrasound had no clear trend compared to continuous ultrasound.

**Table 1:** PE values at each pulsed setting: pulse length (0.1–1 s), pulse interval (0.1–1 s).

Pulsed	Pulse interval (s)									
Pulse length (s)	**0.1**	**0.2**	**0.3**	**0.4**	**0.5**	**0.6**	**0.7**	**0.8**	**0.9**	**1.0**
0.1	−100.0 ± 0.0	−100.0 ± 0.0	−100.0 ± 0.0	−100.0 ± 0.0	−100.0 ± 0.0	−100.0 ± 0.0	−100.0 ± 0.0	−100.0 ± 0.0	−100.0 ± 0.0	−100.0 ± 0.0
0.2	−62.4 ± 1.9	−61.4 ± 1.7	−63.9 ± 0.6	−60.4 ± 2.3	−64.9 ± 1.1	−63.1 ± 1.5	−62.7 ± 1.5	−62.1 ± 1.7	−63.8 ± 1.0	−61.8 ± 0.8
0.3	−43.0 ± 0.6	−43.1 ± 2.1	−47.7 ± 0.8	−42.1 ± 0.9	−44.1 ± 0.8	−41.7 ± 0.7	−41.0 ± 1.7	−48.3 ± 0.2	−44.9 ± 1.0	−41.0 ± 2.0
0.4	−27.7 ± 0.8	−28.9 ± 1.0	−30.3 ± 1.0	−27.8 ± 0.8	−32.7 ± 1.8	−32.1 ± 1.4	−30.2 ± 0.9	−30.9 ± 3.0	−29.8 ± 2.6	−29.2 ± 1.3
0.5	−26.5 ± 1.3	−31.3 ± 1.8	−30.6 ± 0.6	−27.5 ± 1.8	−27.8 ± 1.8	−24.3 ± 2.1	−24.5 ± 1.8	−20.4 ± 1.3	−34.1 ± 1.7	−32. 7 ± 1.8
0.6	−27.6 ± 0.9	−32.7 ± 1.5	−13.9 ± 0.8	−26.3 ± 1.8	−12.6 ± 1.7	−26.06 ± 0.9	−23.3 ± 1.4	−24.1 ± 1.8	−27.8 ± 0.9	−26.8 ± 1.0
0.7	−20.5 ± 1.9	−20.4 ± 2.3	−27.4 ± 1.4	−20.4 ± 1.0	−24.8 ± 1.7	−23.4 ± 1.5	−25.9 ± 1.2	−17.2 ± 1.3	−20.6 ± 0.3	−12.4 ± 1.4
0.8	−20.3 ± 0.9	−16.9 ± 1.7	−12.8 ± 1.7	−17.9 ± 1.7	0.3 ± 1.9	−2.6 ± 1.7	−16.6 ± 1.4	−12.2 ± 4.1	−12.5 ± 3.5	−16.7 ± 2.9
0.9	−24.2 ± 2.0	−26.1 ± 1.8	−11.5 ± 1.3	−17.2 ± 2.5	−24.1 ± 1.5	−21.9 ± 0.0	−20.1 ± 2.0	−21.8 ± 1.8	−25.9 ± 2.0	−27.8 ± 0.3
1.0	−7.3 ± 2.3	−9.2 ± 2.4	−8.5 ± 1.8	−12.0 ± 2.0	−10.2 ± 1.4	−5.3 ± 0.3	−10.6 ± 1.0	−1.6 ± 0.6	0.4 ± 1.8	0.4 ± 0.4

**Table 2:** PE values at each pulsed setting: pulse length (1–9 s), pulse interval (1–9 s).

Pulsed	Pulse interval (s)								
Pulse length (s)	**1.0**	**2.0**	**3.0**	**4.0**	**5.0**	**6.0**	**7.0**	**8.0**	**9.0**
1.0	0.5 ± 0.2	4.1 ± 1.4	−2.7 ± 1.3	3.4 ± 0.4	−3.6 ± 0.7	1.6 ± 1.5	5.8 ± 1.7	3.4 ± 0.3	11.6 ± 1.3
2.0	−7.4 ± 0.5	10.7 ± 1.4	4.5 ± 1.7	7.6 ± 1.2	9.9 ± 1.3	8.9 ± 0.8	11.3 ± 0.9	17.8 ± 1.9	6.4 ± 1.7
3.0	−9.3 ± 0.9	2.2 ± 0.9	12.3 ± 1.0	3.9 ± 0.8	1.2 ± 0.7	7.1 ± 1.5	4.9 ± 0.8	−1.8 ± 1.5	1.8 ± 1.4
4.0	−9.4 ± 1.0	−14.3 ± 0.9	−16.6 ± 1.0	5.3 ± 1.8	−10.1 ± 1.8	−9.6 ± 0.6	−5.5 ± 0.9	−5.4 ± 0.6	−12.2 ± 0.5
5.0	−5.6 ± 0.9	−13.9 ± 0.6	−12.2 ± 1.9	−6.7 ± 0.0	8.8 ± 1.4	6.8 ± 0.8	9.1 ± 1.4	7.1 ± 1.3	10.5 ± 1.5
6.0	−10.9 ± 1.7	−7.4 ± 0.9	4.9 ± 1.3	−0.9 ± 1.2	−2.2 ± 0.8	9.7 ± 0.3	−3.5 ± 1.0	0.7 ± 1.4	−10.4 ± 1.8
7.0	−6.6 ± 0.8	−19.5 ± 0.7	−18.4 ± 0.8	−14.2 ± 0.4	−13.8 ± 0.7	−7.9 ± 1.0	10.0 ± 0.6	−5.0 ± 1.7	−7.3 ± 0.8
8.0	−9.5 ± 0.9	−20.0 ± 1.9	−18.8 ± 2.0	−2.5 ± 1.3	−12.1 ± 1.9	−14.3 ± 0.9	−3.4 ± 0.4	11.2 ± 1.5	−5.0 ± 0.4
9.0	−19.5 ± 1.5	−18.6 ± 0.9	−23.2 ± 0.9	−24.3 ± 1.7	−20.6 ± 0.8	−12.7 ± 0.8	−3.3 ± 0.7	−8.3 ± 0.7	11.0 ± 0.2

**Figure 1:**
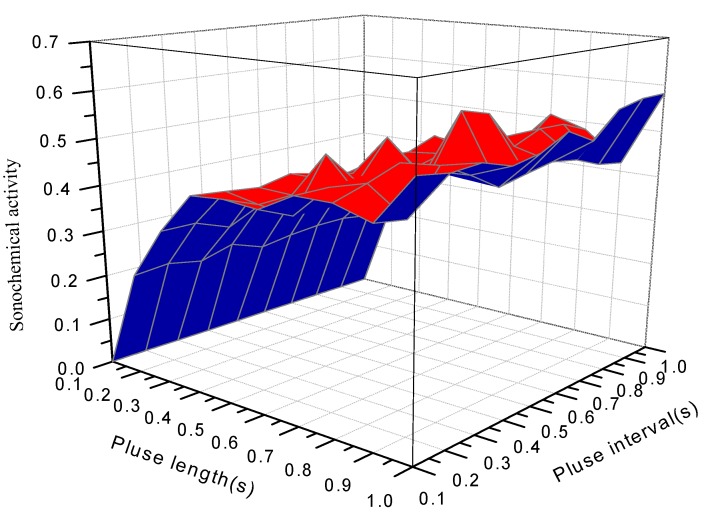
Influence of pulse length (0.1–1 s) and pulse interval (0.1–1 s) on the sonochemical activity.

**Figure 2:**
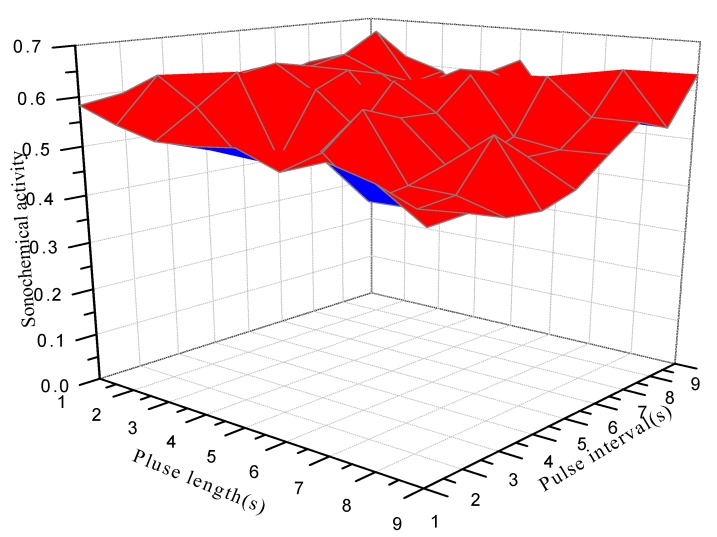
Influence of pulse length (1–9 s) and pulse interval (1–9 s) on the sonochemical activity.

The effects of pulse length and pulse interval on the sonochemical activity are shown in Figure 1, Figure 2. As shown in Figure 1, pulsed ultrasound had no effect when the pulse length was 0.1 s, but sonochemical activity increased with increasing pulse length from 0.1 to 0.4 s. At pulse lengths of 0.4 to 1 s, sonochemical activity had no clear trend. When pulse length varied from 1 to 9 s, sonochemical activity also had no clear trend (Figure 2).

Variance analyses indicated that the main effects of pulse length and pulse interval on sonochemical activity, as well as the interactive effect of pulse length and pulse interval, were significant (*p* < 0.05) under varying conditions of pulse length (1–9 s and 0.1–1 s) and pulse interval (1–9 s and 0.1–1 s). The contribution ratio of pulse length and pulse interval to the deviance sum of squares was 42.6% (pulse length), 17.6% (pulse interval), and 37.3 (pulse length × pulse interval) at pulse lengths of 1–9 s and pulse intervals of 1–9 s. However, their contribution ratio to the deviance sum of squares is 97.4% (pulse length), 0.1% (pulse interval), and 2.1% (pulse length × pulse interval) at pulse lengths of 0.1–1 s and pulse intervals of 0.1–1 s.

**Figure 3:**
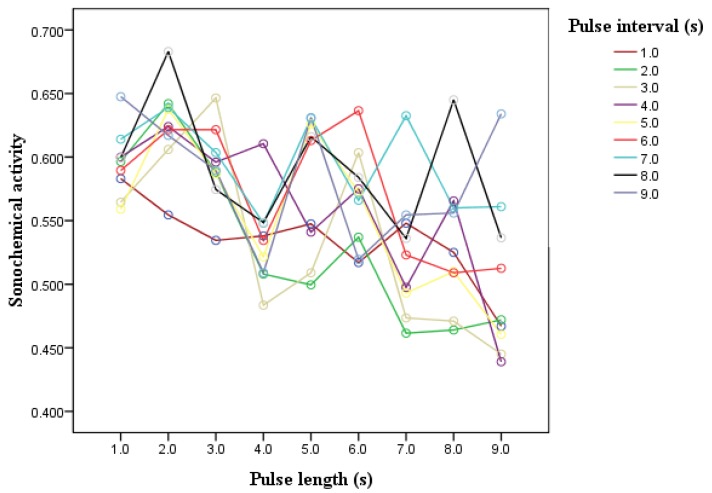
Interaction between pulse length (1–9 s) and pulse interval (1–9 s).

**Figure 4:**
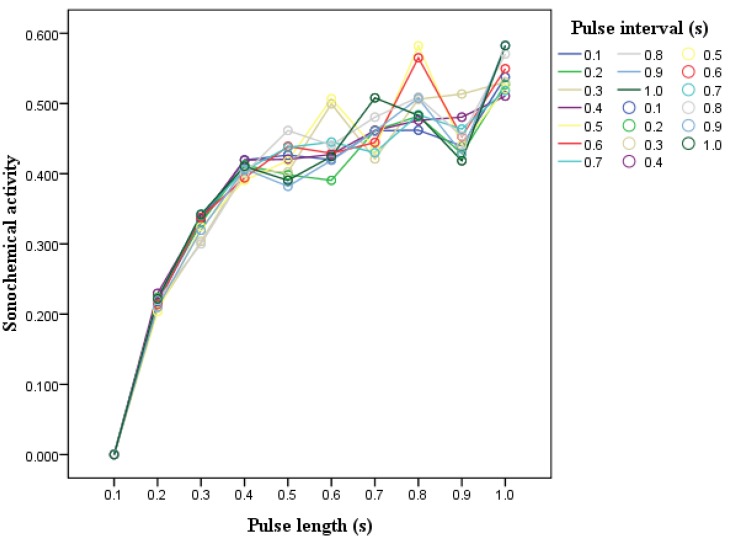
Interaction between pulse length (0.1–1 s) and pulse interval (0.1–1 s).

In addition to the variance analysis of the interaction of pulse length and pulse interval, the interaction was also investigated by interactive lines. Figure 3 shows that the interactive lines cross each other at pulse lengths of 1–9 s and pulse intervals of 1–9 s. Therefore, the interaction of pulse length and pulse interval influenced sonochemical activity. Figure 4 shows that the interactive lines initially almost coincide as the pulse length increases from 0.1 to 0.4 s, then crossed each other as the pulse length increases from 0.5 to 1 s. Thus, the interaction of the pulse length and pulse interval did not influence sonochemical activity as pulse length ranged from 0.1 to 0.4 s, but it influenced sonochemical activity as the pulse length increased from 0.5 to 1 s.

It can be therefore concluded that pulse length was the most important factor of sonochemical activity when pulse length and pulse interval both increased from 0.1 to 1 s. Pulse length, pulse length × pulse interval, and pulse interval were all the important influence factors when both the pulse length and pulse interval ranged from 1 to 9 s.

In the present study, we found that pulsed ultrasound showed different efficacy with the varying pulse length and pulse interval, *i.e.*, the sonochemical activity was sometimes lower, sometimes similar, and sometimes higher compared to that of continuous ultrasound. These results are different from those of previous studies [16,17,21], which reported that at higher power, the efficacy of pulsed ultrasound was weaker than that of continuous ultrasound, whereas at lower power, the efficacy of pulsed ultrasound was greater than or similar to that of continuous ultrasound. Our results can be explained [16]: a pulse does not produce a chemical reaction immediately but needs some time, denoted as the “activation time” τ_1_, to form gas bubbles of suitable size. After the pulse, the bubbles disappear with a characteristic time, the “deactivation time” τ_2_. If the interval T_off_ between the pulses is longer than τ_2_, the following pulse has to activate the solution anew. However, if T_off_ < τ_2_, the subsequent pulse can still act on the bubbles formed by the preceding pulse and in this way be more chemically efficient. If the pulse length T_on_ is shorter than τ_1_, no chemical effects occur.

Pulse ultrasound had no sonochemical effect when the pulse length was 0.1 s, and the reason may be that in this case pulse length is shorter than activation time. These results are different from those of other studies. For example, Deojay [20] found that sonochemical effects occurred when the pulse length was 0.03, 0.06, and 0.1 s in aqueous octylbenzene sulfonate solutions. Tuziuti *et al*. [19] found that sonochemical effects also occurred when the pulse length was 0.006 and 0.06 s in aqueous solution of potassium iodide. The differences may be due to the different activation times required for the different ultrasonic equipment.

The lower efficacy of pulsed ultrasound when compared to continuous ultrasound as the pulse length varied from 0.2 to 1 s can be explained by the fact that not all bubbles have enough time to grow to be active bubbles, which causes a reduced number of cavitation events.

However, the sonochemical activity of pulsed ultrasound had no clear trend as the pulse length increased from 1 to 9 s. The higher or lower efficacy compared to that of continuous ultrasound may be mainly caused by the ratio of T_on_ and T_off_. Sometimes the sonochemical activity was higher than that of continuous ultrasound, which may be attributed to the residual pressure amplitude during the pulse-off time and to the spatial enlargement of active reaction sites [19]. Sometimes the sonochemical activity was lower than continuous ultrasound, which may be because the coalescence is so fast that chemical activity is lost very quickly during the pulse-off time and cannot be topped-up to the level obtained upon continuous irradiation. 

### 2.2. The Effect of Treatment Time on the Pulse Enhancement and Sonochemical Activity

The above analysis was carried out at a fixed treatment time of 20 min. However, treatment time is also an important factor for PE and sonochemical activity. Taking two pulsed modes, 2 s/2 s and 3 s/2 s (T_on_/T_off_) as examples, Table 3 shows that the PE of the 2 s/2 s pulse type increased with time ranging from 4 to 16 min. The PE of the 3 s/2 s pulse type initially decreased, then increased with time ranging from 4 to 16 min. The PE of the 3 s/2 s pulse type was higher than that of the 2 s/2 s pulse type at a treatment time of 4 min, was the same as that of the 2 s/2 s pulse type at a treatment time of 12 min, and was lower than that of the 2 s/2 s pulse type at a treatment time of 16 min.

**Table 3:** The PE values of 3 s/2 s pulsed type and 2 s/2 s pulsed type at different time (4–16 min).

Pulsed mode (T_on_/T_off_)	PE (%)			
	4 min	8 min	12 min	16 min
3 s/2 s	17.7 ± 1.4	5.9 ± 0.3	13.4 ± 0.2	18.3 ± 2.4
2 s/2 s	−2.0 ± 0.8	3.6 ± 0.6	13.3 ± 1.3	23.3 ± 1.0

**Figure 5:**
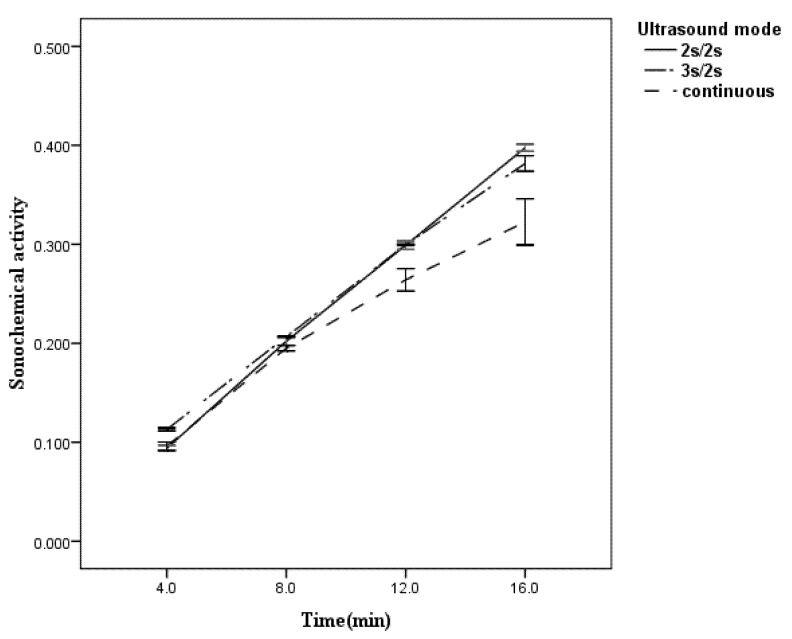
Influence of ultrasound treatment time on the sonochemical activity.

From the trend lines of sonochemical activity under the 2 s/2 s pulse type, the 3 s/2 s pulse type, and continuous ultrasound (Figure 5), it was found that the reaction rate of sonochemical activity was almost constant with the 2 s/2 s pulse type, slightly decreased with the 3 s/2 s pulse type, and significantly decreased with the continuous ultrasound. It can be concluded, therefore, that the 3 s/2 s pulse type is a good choice when the treatment time is short and the 2 s/2 s pulse type is a good choice when the treatment time is long. A similar phenomenon was observed previously in the degradation of octylbenzene sulfonate (OBS), which showed a decrease in the rate of OBS degradation over long-term sonolysis under continuous ultrasound but not under pulsed ultrasound [20]. The author thought the byproducts formed competed with the parent compounds, slowing the reaction rate under continuous ultrasound, but that the same effect did not occur with the pulsed ultrasound because of the pulse-off time was long enough to absorb the parent compound to the gas/solution interface of the cavitation bubbles. Based on our results, the main reason is thought to be a decrease in the performance of the transducer with time as a result of the heat accumulation of the transducer under continuous ultrasound and the normal performance of the transducer with time because of the releasing of heat of the transducer during the pulse-off time under pulsed ultrasound.

## 3. Experimental

### 3.1. Chemicals

Purified water was purchased from Hangzhou Wahaha Group Co., Ltd. (Hangzhou, China). Analytical-grade potassium iodide (purity ≥ 99.0%) was purchased from Sinopharm Chemical Reagent Co. Ltd. (Shanghai, China).

### 3.2. Ultrasound Treatments

Ultrasound treatments were carried out with a probe ultrasonic processor (Scientz-IID, Ningbo Scientz Biotechnology Co., Ningbo, China). Some parameters of the probe ultrasonic processor are as follows: the highest input electric power is 950 W, frequency is 20 kHz, and the diameter of horn microtip is 10 mm. A 0.2 M KI solution was prepared in a volumetric flask. The solution was added to brown glass tubes (3 cm diameter × 12 cm long), saturated by air, then the tubes with solution were immersed in a low-temperature thermostatic water bath ranging from −10 to 100 °C (DC-1006, Safe Corporation, Ningbo, China) to maintain a constant temperature. The solution was then treated by ultrasound. The general ultrasound conditions were as follows: the probe was placed 1 cm from the top surface of the extraction cell. The liquid height measured from the horn microtip to the tube bottom was 4 cm. The temperature was 5 °C. The ultrasound power absorbed by the liquid was determined by calorimetric method, which based on monitoring the increase of temperature due to conversion of the ultrasonic energy into heat. The temperature increase was followed by a type-K thermocouple after switching on the ultrasonic generator. The ultrasound intensity was 2.7 W/cm^2^. The ultrasound treatment time was 20 min. The ultrasound treatment time refers to the actual working time that is equal to the continuous ultrasound treatment time; the total pulsed ultrasound treatment time was longer than that of the continuous ultrasound treatment, as shown in Equation (1).

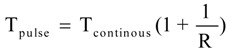
(1)
where R is the ratio of T_on_/T_off_ for the pulse wave. Pulsed mode was T_on_ = 0.1~9 s and T_off_ = 0.1~9 s. The treated solution was stored at 4 °C for further spectrophotometric analyses.

### 3.3. Analytical Method of Sonochemical Activity and Pulse Enhancement

The sonochemical activity of the pulsed ultrasound was measured by the oxidation of potassium iodide [19]. Sonochemical activity is directly proportional to the absorbance of I_3_^−^ under pulsed ultrasound treatment, which was expressed as the absorbance of I_3_^−^. The glass tubes were filled with an aqueous solution of 0.2 M potassium iodide prepared from purified water and the solution was under saturated air. The oxidation of I^−^ provides I_2_, and I_3_^−^ is produced by the reaction of I_2_ and I^−^. The absorbance of I_3_^−^ was measured with a spectrophotometer (Shimadzu UV-2550) at 350 nm. The absorbance value was measured under different ultrasound treatment conditions. To compare the sonochemical activity of the pulsed ultrasound to continuous ultrasound, pulse enhancement (PE) was calculated as

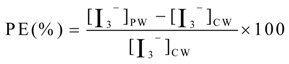
(2)
where [I_3_^−^]_PW_ is the absorbance of the formation of I_3_^−^ during pulsed ultrasound treatment and [I_3_^−^]_CW_ is the absorbance of the formation of I_3_^−^ during continuous ultrasound treatment.

### 3.4. Experimental Design

A factorial design was used to investigate the main and interactive effects of two independent variables: pulse length and pulse interval. When pulse length and pulse interval varied from 0.1 to 1 s, respectively, a factorial design of 10^2^ was used. When pulse length and pulse interval varied from 1 to 9 s, respectively, a factorial design of 9^2^ was used. The complete design thus consisted of 100 and 81 experimental points for intervals from 0.1 to 1 s and 1 to 9 s, respectively. The 181 sets of experiments were performed in a random order.

### 3.5. Statistical Analysis

Each treatment was replicated three times. The results were expressed as mean ± SD. According to the experimental design used, the mathematical description consists of two main effects and a two-factor interaction. The significance was tested using analysis of variance using SPSS16.0. Response surfaces were developed using Origin7.5.

## 4. Conclusions

In conclusion, pulsed ultrasound had no effect when the pulse length was 0.1 s. However, the sonochemical activity of pulsed ultrasound decreased compared to that of continuous ultrasound as the pulse length and pulse interval increased from 0.1 to 1 s, and increased or decreased compared to continuous ultrasound when the pulse length and pulse interval varied from 1 to 9 s. The increase or decrease of sonochemical activity of pulsed ultrasound had no clear trend compared with that of continuous ultrasound. The sonochemical activity of some pulsed types was constant or slightly decreased with time, but the sonochemical activity of continuous ultrasound significantly decreased with time. Although the changes in sonochemical activity of pulsed ultrasound were studied for a fixed time, the changes with time only were studied under two special conditions. The changes with time and mechanism of the changes need further investigation. 

## References

[1] Lakshmisha C.N., Ravishankar G., Ninan C.O., Mohan T.K., Gopal S. (2008). Effect of freezing time on the quality of Indian mackerel (*Rastrelliger kanagurta*) during frozen storage. J. Food Sci..

[2] Cameron M., McMaster L.D., Britz T.J. (2009). Impact of ultrasound on dairy spoilage microbes and milk components. Dairy Sci. Technol..

[3] Pingret D., Fabiano-Tixier A.S., Petitcolas E., Canselier J.P., Chemat F. (2011). First investigation on ultrasound-assisted preparation of food products: Sensory and physicochemical characteristics. J. Food Sci..

[4] Chemat F., Khan M.K. (2011). Applications of ultrasound in food technology: Processing, preservation and extraction. Ultrason. Sonochem..

[5] Zhang Y., Zhang W., Liao X. (2010). Degradation of diazinon in apple juice by ultrasonic treatment. Ultrason. Sonochem..

[6] Zhang Y., Xiao Z., Chen F. (2010). Degradation behavior and products of malathion and chlorpyrifos spiked in apple juice by ultrasonic treatment. Ultrason. Sonochem..

[7] Pan Z.L., Qu W.J., Ma H.L., Atungulu G.G., Mchugh T.H. (2011). Continuous and pulsed ultrasound-assisted extractions of antioxidants from pomegranate peel. Ultrason. Sonochem..

[8] Skirtenko N., Tzanov T., Gedanken A., Rahimipour S. (2010). One-step preparation of multifunctional chitosan microspheres by a simple sonochemical method. Chem. A Eur. J..

[9] Zúñiga R.N., Kulozik U., Aguilera J.M. (2011). Ultrasonic generation of aerated gelatin gels stabilized by whey protein β-lactoglobulin. Food hydrocolloid..

[10] Nomura H., Koda S., Yasuda K., Kojima Y. (1996). Quantification of ultrasonic intensity based on the decomposition reaction of porphyrin. Ultrason. Sonochem..

[11] Rong L., Kojima Y., Koda S., Nomura H. (2001). Simple quantication of ultrasonic intensity using aqueous solution of phenolphthalein. Ultrason. Sonochem..

[12] Hu Y., Zhang Z., Yang C. (2008). Measurement of hydroxyl radical production in ultrasonic aqueous solutions by a novel chemiluminescence method. Ultrason. Sonochem..

[13] Adekunte A.O., Tiwari B.K., Cullen P.J., Scannell A.G.M., O’Donnell C.P. (2010). Effect of sonication on colour, ascorbic acid and yeast inactivation in tomato juice. Food Chem..

[14] Valdramidis V.P., Cullen P.J., Tiwari B.K., O’Donnell C.P. (2010). Quantitative modelling approaches for ascorbic acid degradation and non-enzymatic browning of orange juice during ultrasound processing. J. Food Eng..

[15] Rodríguez-Rojo S., Visentin A., Maestri D., Cocero M.J. (2012). Assisted extraction of rosemary antioxidants with green solvents. J. Food Eng..

[16] Gutiérrez M., Henglein A. (1990). Chemical action of pulsed ultrasound: Observation of an unprecedented intensity effect. J. Phys. Chem..

[17] Dekerckheer C., Bartik K., Lecomte J.P., Reisse J. (1998). Pulsed Sonochemistry. J. Phys. Chem. A.

[18] Casadonte D.J., Flores M., Petrier C. (2005). Enhancing sonochemical activity in aqueous media using power-modulated pulsed ultrasound: An initial study. Ultrason. Sonochem..

[19] Tuziuti T., Yasui K., Lee J., Kozuka T., Towata A., Iida Y. (2008). Mechanism of enhancement of sonochemical-reaction efficiency by pulsed ultrasound. J. Phys. Chem. A.

[20] Deojay D.M., Sostaric J.Z., Weavers L.K. (2011). Exploring the effects of pulsed ultrasound at 205 and 616 kHz on the sonochemical degradation of octylbenzene sulfonate. Ultrason. Sonochem..

[21] Mitome H., Hatanaka S. (1990). Chemical action of pulsed ultrasound: Observation of an unprecedented intensity effect. J. Phys. Chem..

